# Studying Synaptically Evoked Cortical Responses *ex vivo* With Combination of a Single Neuron Recording (Whole-Cell) and Population Voltage Imaging (Genetically Encoded Voltage Indicator)

**DOI:** 10.3389/fnins.2021.773883

**Published:** 2021-10-27

**Authors:** Jinyoung Jang, Mei Hong Zhu, Aditi H. Jogdand, Srdjan D. Antic

**Affiliations:** Department of Neuroscience, Institute for Systems Genomics, University of Connecticut School of Medicine, Farmington, CT, United States

**Keywords:** VSFP, Butterfly, ArcLight, Archon1, di-4-ANEPPS

## Abstract

In a typical electrophysiology experiment, synaptic stimulus is delivered in a cortical layer (1–6) and neuronal responses are recorded intracellularly in individual neurons. We recreated this standard electrophysiological paradigm in brain slices of mice expressing genetically encoded voltage indicators (GEVIs). This allowed us to monitor membrane voltages in the target pyramidal neurons (whole-cell), and population voltages in the surrounding neuropil (optical imaging), simultaneously. Pyramidal neurons have complex dendritic trees that span multiple cortical layers. GEVI imaging revealed areas of the brain slice that experienced the strongest depolarization on a specific synaptic stimulus (location and intensity), thus identifying cortical layers that contribute the most afferent activity to the recorded somatic voltage waveform. By combining whole-cell with GEVI imaging, we obtained a crude distribution of activated synaptic afferents in respect to the dendritic tree of a pyramidal cell. Synaptically evoked voltage waves propagating through the cortical neuropil (dendrites and axons) were not static but rather they changed on a millisecond scale. Voltage imaging can identify areas of brain slices in which the neuropil was in a sustained depolarization (plateau), long after the stimulus onset. Upon a barrage of synaptic inputs, a cortical pyramidal neuron experiences: (a) weak temporal summation of evoked voltage transients (EPSPs); and (b) afterhyperpolarization (intracellular recording), which are not represented in the GEVI population imaging signal (optical signal). To explain these findings [(a) and (b)], we used four voltage indicators (ArcLightD, chi-VSFP, Archon1, and di-4-ANEPPS) with different optical sensitivity, optical response speed, labeling strategy, and a target neuron type. All four imaging methods were used in an identical experimental paradigm: layer 1 (L1) synaptic stimulation, to allow direct comparisons. The population voltage signal showed paired-pulse facilitation, caused in part by additional recruitment of new neurons and dendrites. “*Synaptic stimulation*” delivered in L1 depolarizes almost an entire cortical column to some degree.

## Introduction

Modern neuroscience aims to develop a structure–function model of nervous system organization that would allow mechanistic linking of brain and behavior. A necessary but not sufficient foundation is a *connectome*, a description of structural connections between nerve cells (Swanson and Lichtman, 2016). The current connectome approaches often rely solely on structural mapping and therefore cannot determine, evaluate, or gauge the functionality of reported synaptic connections. The presence of structural synaptic elements (i.e., presynaptic bouton adjacent to postsynaptic process) alone does not confirm functional connections. It is therefore crucial to extend current connectome mapping approaches to incorporate functional probing of synaptic connections among defined neuronal populations (Nakajima and Baker, 2018).

Mapping the connectivity of pairs of neocortical excitatory neurons is limited to the few neurons selected for whole-cell recording (Markram et al., 1997; Holmgren et al., 2003) and cannot address what occurs when a much larger ensemble of neurons is stimulated. Voltage-sensitive dye (VSD) imaging signals can address subthreshold (synaptic) depolarizations in a much larger ensemble of neurons to explore functionally dependent areas, activity in supragranular and infragranular cortical laminas, activity in neighboring cortical columns, the spread of depolarization waves in respect to speed and direction, cortical oscillations, as well as the plasticity of cortical maps induced by alterations in sensory experience (Prechtl et al., 1997; Petersen and Sakmann, 2001; Petersen et al., 2003; Grinvald and Hildesheim, 2004; Huang et al., 2010; Song et al., 2018). Genetically encoded voltage indicators (GEVIs) show a great promise for further improving traditional voltage imaging methods and extracting new information for deciphering cortical circuits (Storace et al., 2016; Platisa and Pieribone, 2018; Knopfel and Song, 2019). First, the use of GEVIs eliminates the problems with toxic and pharmacological effects of VSDs (Mennerick et al., 2010; Grandy et al., 2012; Kuhn and Roome, 2019). Second, GEVIs can be selectively expressed in one neuron cell type [e.g., layer 2/3 (L2/3) neocortical pyramidal neuron], so that the recorded optical signals are not contaminated by activities of other cell types (Empson et al., 2015).

In live animal recordings (*in vivo*), physiological signals are influenced by heart rate, breathing, motion artifacts, sensory inputs, neuromodulatory inputs, and brain states. Brain slices, on the other hand, are better suited to study the elementary properties of brain circuits (e.g., membrane excitability, synaptic plasticity, and neuromodulation), as well as for accessing the deeper regions in the brain (e.g., neocortical layer 5). In the present study, we used acute brain slices to combine whole-cell recordings from pyramidal neurons and multi-site voltage imaging from the cortical neuropil surrounding a pyramidal cell of interest. Each of the four voltage indicators (chi-VSFP, ArcLightD, Archon1, and di-4-ANEPPS) provided some new and unique optical features related to the sensitivity, response speed, expression pattern, excitation/emission spectra and cell-type specificity. For example, in some brain slices, the GEVI [chimeric voltage sensitive fluorescent protein (chi-VSFP)] was exclusively expressed in excitatory pyramidal neurons (genetically), while in other brain slices of the current study, the VSD (di-4-ANEPPS) was applied in the extracellular solution to stain cellular membranes. An extracellular application of lipophilic VSDs indiscriminately stains all plasma membranes (cell body, dendrites, dendritic spines, axons, and synaptic terminals), belonging to all pyramidal cells, GABAergic interneurons, L4 stellate cells, astrocytes, microglia, oligodendrocytes, and epithelia. Both sets of labeled brain slices, chi-VSFP and di-4-ANEPPS, were stimulated and imaged in an identical manner, to allow comparisons of synaptically evoked voltage waveforms.

## Materials and Methods

### Genetically Encoded Voltage Indicators and Dyes

The transgenic animal line, “*PAN-GEVI*,” was kindly donated by Thomas Knopfel (Imperial College London, United Kingdom). ArcLightD was kindly provided by Jelena Platisa and Vincent Pieribone (Yale University, New Haven, CT, United States). Archon1 was kindly provided by Kiryl Piatkevich and Ed Boyden (MIT, Boston, MA, United States). di-4-ANEPPS was purchased from Thermo Fisher Scientific (cat. D1199).

### Animals

Black C57BL/6 mice of either sex were used for the intracerebroventricular (ICV) injections of GEVIs packed in several variants of adeno-associated virus (AAV) backbones (animal ages P0.5–P1), according to the animal protocols approved by the UConn Health Institutional Animal Care and Use Committee (IACUC). In contrast, the PAN-GEVI mice expressed chi-VSFP in all cortical pyramidal neurons (CaMK2A-tTA;tetO-chiVSFP) at birth. All animals were housed in standard conditions with free access to food and water, in a 50% dark/light cycle.

### Intracerebroventricular Injections

Adeno-associated viruses containing the sequence of GEVIs of interest were mixed with trypan blue solution, and loaded into a Hamilton syringe, attached to a Narishige mechanical micromanipulator (NMN-21). Newborn (P0.5–P1) mice of either sex were cold anesthetized by placing on ice for a couple of minutes, and then positioned on the pad below the Hamilton syringe, so that the needle touches the skull surface at a location approximately 0.25 mm lateral to the sagittal suture and 0.50–0.75 mm rostral to the neonatal coronary suture. The needle was then carefully inserted into the skull 2–3 mm deep *via* a micromanipulator. A volume of 1–2 μl of solution was slowly injected (for ∼30 s with several 3–5 s pauses) into the lateral ventricle. After the injections, bright white light was shone through the skull to reveal trypan blue-filled ventricles, and mice were placed on a heated pad to recover prior to returning them to the breeding cage.

### Optical Filters for Voltage Imaging

ArcLightD and chi-VSFP were excited using a 470 nm light emitting diode, LED (pE, CoolLED, Andover, United Kingdom). Excitation filter: 480/40 nm, dichroic 510 nm, and emission filter: 535/50 nm. Archon1 and di-4-ANEPPS were excited with a metal halide lamp (Lumen 200, Prior Scientific) or LED (pE, CoolLED, United Kingdom). For di-4-ANEPPS, the excitation filter: 520/60 nm, dichroic 570 nm, and emission filter: 610 nm long-pass. For Archon1, we used the excitation filter: 605/30 nm, dichroic: 640 nm, and emission filter: 665 nm long pass.

### Brain Slice, Electrophysiology, and Voltage Imaging

Intracerebroventricular-injected and transgenic mice (P30–P90) were anesthetized with isoflurane inhalation, decapitated, and brains were extracted with the head immersed in ice-cold artificial cerebrospinal fluid (ASCF). ACSF contained (in mM) 125 NaCl, 26 NaHCO_3_, 10 glucose, 2.3 KCl, 1.26 KH2PO_4_, 2 CaCl_2_, and 1 MgSO_4_. Coronal slices (300 μm) were cut from the frontoparietal cortex, incubated at 37°C for 30 min and then at room temperature prior to experimental recordings. All experimental measurements were performed at 32–34°C. Acute brain slices were transferred to an Olympus BX51WI upright microscope or Zeiss Axioskop 2F, and perfused with aerated (5% CO_2_/95% O_2_) ACSF at 32–34°C. Whole-cell patch clamp recordings were done in current clamp configuration, where electrical signals were amplified with Multiclamp 700B and digitized with two input boards: (1) Digidata Series 1400A (Molecular Devices, Union City, CA, United States) and (2) Neuroplex (RedShirtImaging, Decatur, GA, United States). The extracellular stimulation (often termed “*synaptic stimulation*” in the literature) was achieved by a computer-controlled stimulus isolation unit, IsoFlex (A.M.P.I., Jerusalem, Israel). The stimulation electrodes were pulled from borosilicate glass filament (1.5 mm outer diameter; 0.8 mm inner diameter; resistance ∼7 MΩ) filled with ACSF, and positioned in superficial cortical layers. Triplets of extracellular (“synaptic”) shocks at 8.3 and 83 Hz were delivered in the same optical recording sweep, separated by a 1.1 s interval. The duration of a typical optical sweep was 3 s (shutter open time = 3 s). Optical traces were repeated every 15–20 s. For excitation of brain slices, metal halide lamp (Lumen 200, Prior Scientific) was used or LED (pE, CoolLED, United Kingdom). Optical filters used on brain slices are the same as described in the paragraph “*Optical filters for voltage imaging*.” The intensity of the excitation light was similar between all GEVIs tested on brain slices. Voltage optical signals were sampled with NeuroCCD-SMQ camera (80 × 80 pixel configuration; RedShirtImaging, Decatur, GA, United States), at full-frame sampling interval of 1.02 ms corresponding to a camera frame rate of ∼1,000 Hz.

### Data Analysis

Optical traces were conditioned and analyzed in Neuroplex (RedShirtImaging). Bleach correction was done by subtracting an exponential fit from the optical trace, or by subtracting a real optical recording obtained in the same visual field with stimulus omitted (a “no stimulus trial”). Temporal averaging (*n* = 4 sweeps), spatial averaging (21–37 pixels), low-pass Gaussian filter with a 77 Hz cutoff, and high-pass Tau filter (10), unless stated otherwise. The optical signal amplitude was quantified as difference between the baseline before the synaptic stimulus and the Peak of optical transient. Optical signal amplitudes are expressed as ΔF/F, where F represents the resting fluorescence intensity at the beginning of the optical trace (baseline), and ΔF represents the intensity change from the baseline fluorescence during the biological signal. No additional corrections of F were used for the voltage imaging data. Color-coded amplitude page display of the voltage imaging data was generated in *Neuroplex* using the “frame subtraction” command, in which one baseline frame is subtracted from the frame under the investigation. To improve the quality of optical signals in the page display, time binning (2 or 3), and spatial processing LP filter: 3 × 3 mean was used at iteration = 3, to smooth out the boundaries between the red, yellow, green and blue amplitude areas. Data organization, plotting, and statistical testing (unpaired Student’s *t*-test) were done in Excel.

## Results

### Trains of Action Potentials in Single Neurons

**FIGURE 1:**
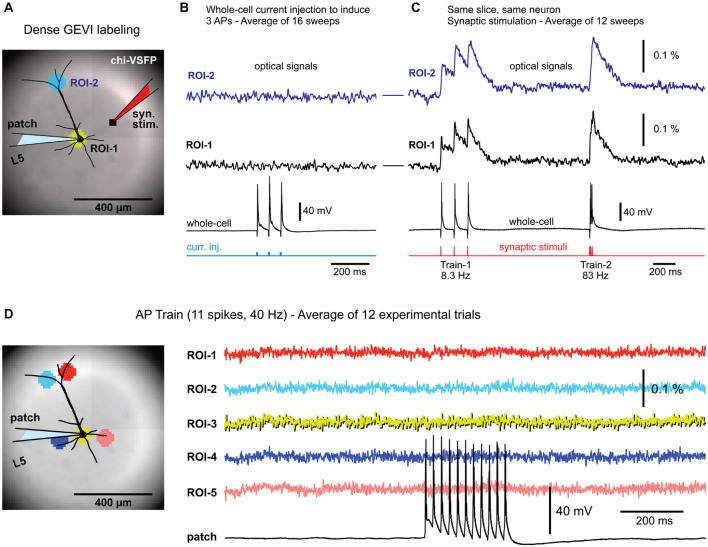
Background fluorescence obscures optical signals from a single-neuron. **(A)** Brain slice from transgenic GEVI animal (chi-VSFP). One L5 neuron is patched and stimulated to generate three action potentials (16 Hz). **(B)** Optical signals from the cell body (ROI-1), or optical signals from the apical tuft area (ROI-2), show no AP-associated voltage transients, despite a 16-trial temporal averaging. **(C)** In contrast, simple (low intensity) synaptic stimulations regularly evoke optical depolarization signals (ROI-1 and ROI-2). **(D)** Left (image): five ROIs are positioned in the apical and basilar regions of a patched pyramidal cell. Right (traces): trains of 11 APs, at 40 Hz, failed to produce any discernable optical signals in the selected ROIs, or anywhere else in the visual field.

We tested if driving one pyramidal neuron to fire action potentials and simultaneously recording voltage in the surrounding neuropil, would produce useful physiological data that can be used for studying cortical physiology. In theory, two mechanisms may produce voltage signals in a neuropil surrounding cortical pyramidal cells. First, AP backpropagation into the dendrite may produce dendritic optical voltage signals (Antic, 2003). Such optical signals should be the strongest in locations where many individual branches overlap, i.e., project onto the same detector, and generate a compound optical signal (Figure 1A). Second, during a cell body firing episode, APs invade axons, and AP-mediated evoked voltage transients (EPSPs) are generated in dendrites of the postsynaptic partners (Feldmeyer and Sakmann, 2000). If these postsynaptic dendrites are within the imagining area (Figure 1A), while presynaptic APs are produced in a regular manner (i.e., evoked by patterned stimuli) and averaged across many experimental trials, it might be possible to extract a compound EPSP signal in brain slice experiments, and study cortical connectome, for example.

We tested the existence of any voltage-imaging signatures occurring when individual neurons experience: (a) backpropagating APs; or (b) when individual neurons cause AP-mediated EPSP signals in the neighboring cells. Brain slices were prepared from transgenic animals expressing a type of a GEVI called chi-VSFP-Butterfly (Mishina et al., 2014). In these animals, all cortical pyramidal neurons express GEVI, which increases our chances of capturing population voltage responses (Zhu et al., 2021). One pyramidal cell per brain slice was patched (whole-cell) in a current clamp mode. Optical signals were sampled in the area surrounding the patched cell (Figure 1A), while trains of APs were evoked by brief current pulses injected into the cell body (Figure 1B, current injection). Electrophysiology-optical trials were averaged 9–16 times to increase chances of capturing small optical signals. This extensive averaging failed to produce any discernable bAP-associated optical signal in the basilar or apical region of interest (ROI-1 or ROI-2, respectively). However, in the same experiment in which somatic APs failed to produce optical signals, the synaptic stimulation, on the other hand, resulted in robust optical responses in the very same ROIs (Figure 1C), assuring: (1) the viability of the actual brain slice preparation, (2) presence of functional GEVI in the neuronal membranes, and (3) GEVI’s sensitivity to biological electrical signals. This sequence of two steps, step 1 = direct current injection + voltage imaging, followed by step 2 = synaptic stimulation + voltage imaging, was conducted in four pyramidal neurons, with an identical outcome. Each time, somatic AP failed to produce optical signals (Figure 1B), while the synaptic stimulation succeeded in the generation of optical signals (Figure 1C). Next, we decided to increase the duration and frequency of AP trains in attempt to generate: (a) stronger and longer bAP-associated dendritic depolarizations and (b) stronger and longer EPSPs in postsynaptic neurons. However, AP trains consisting of 11 spikes at 40 Hz rate also failed to produce any discernable optical signal in the ROI covering the cell body with proximal basal dendritic branches (Figure 1D, ROI-3), or anywhere else in the entire visual field, ROIs 1–5 (*n* = 3).

The GEVI (chi-VSFP) signals recorded at a green emission filter (535/50 nm) have a negative polarity in our raw data records. However, the chi-VSFP optical signals shown in Figure 1 and the following figures have been inverted in display. We feel that inverted GEVI optical signals (positive with depolarization) are more appropriate for presentations.

These data produced four insights. First, strong background fluorescence emanating from thousands of overlapping fluorescent structures (dendrites, axons, and somata) “destroys” optical voltage signals from individual neurons embedded in this environment (Quicke et al., 2019). Those who seek to achieve single-cell resolution in GEVI imaging experiments should arrange for sparse labeling of neurons (Song et al., 2017). Second, when one cell is driven to fire action potentials (Figure 1), some EPSPs are generated in nearby cortical pyramidal neurons (Feldmeyer and Sakmann, 2000; Holmgren et al., 2003). These EPSPs are also lost in the sea of fluorescence despite extensive averaging (Figure 1D). Third, in contrast to the results obtained with driving a single cell to fire APs, a moderate synaptic stimulation (stimulus current intensity = 135 nA, stimulus delivered *via* a monopolar glass microelectrode of a similar size as a standard patch electrode) activates large number of structures (dendrites, axons, somata) and then their combined optical signal easily emerges above the optical noise level (Figure 1C). These data confirmed a well-established fact that the strength of the population voltage signal is in the numbers. Namely, assuming a uniform size of individual dendritic branches and uniform labeling of dendritic membrane, the amplitude of an optical signal will be proportional to the number of activated dendritic branches projecting to the same optical pixel\optical detector. Fourth, synaptic stimulations in this experimental series allowed us to rule out a possibility that our failures to detect optical signal in single cell experiments (Figures 1B,D) were not due to poor experimental conditions. Synaptic stimulation experiments (Figure 1C) were able to rule out the common obstacles in GEVI voltage imaging experiments, such as weak GEVI expression, or weak GEVI sensitivity.

### Voltage Maps of Synaptic Depolarizations in the Neuropil Surrounding the Cortical Pyramidal Cell

A standard electrophysiological experiment involves extracellular (synaptic) stimulation and intracellular recording of EPSPs (Feldmeyer and Sakmann, 2000). Some scientific questions and experimental designs may benefit from knowing the distribution of synaptically evoked potentials in the neuropil surrounding the cortical cell of interest. The voltage waveforms of intracellularly recorded EPSPs may be understood better if one could also observe concomitant voltage waves in dendrites and axons of the neuropil. For example, which set of dendritic branches received the glutamatergic input? Did synaptic inputs activate dendritic branches located in the same cortical column, or synaptic activity occurred in the neighboring columns as well? Was the activity in the neighboring columns weaker or stronger than in the “home” column? How does the somatic EPSP waveform change if one synaptically activates cortical columns on either side of a pyramidal cell? Did synaptic inputs activate dendritic branches located mostly in one cortical layer; if yes, which layer?

**FIGURE 2:**
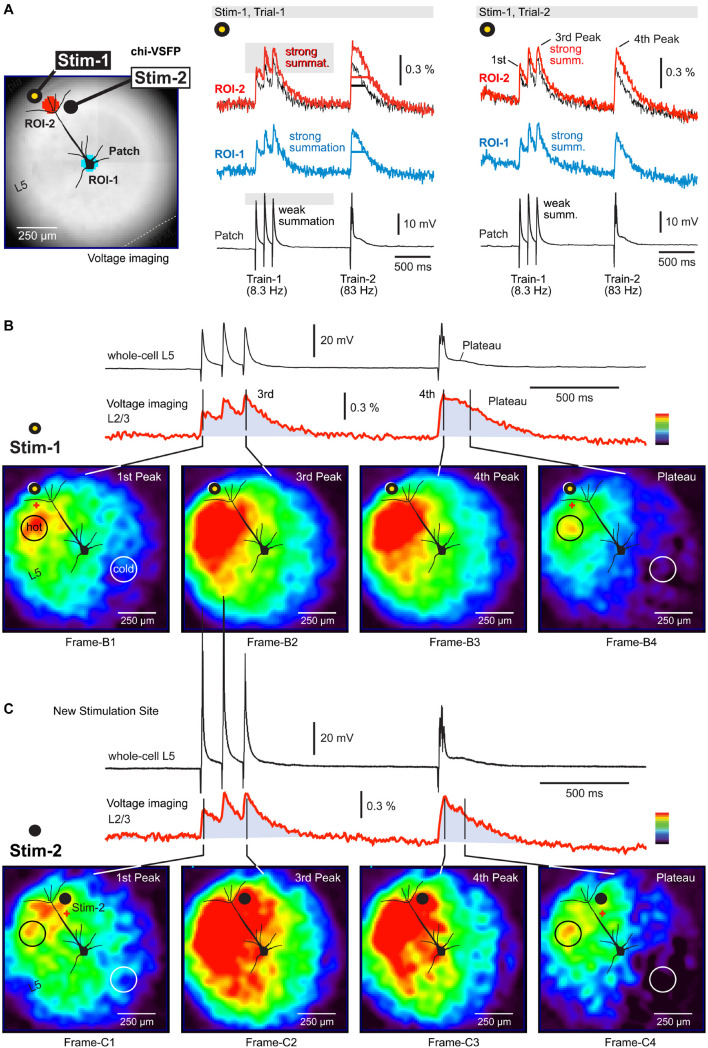
Depolarizations in the neuropil surrounding a neocortical pyramidal neuron. **(A)** A brain slice from the transgenic GEVI (chi-VSFP) animal line. Two stimulation sites are marked by filled dots (yellow and black). Two ROIs are marked by two serrated octagons: turquois and red. Synaptically evoked optical signals (ROIs) and electrical signal (patch), in two consecutive trials (Trial-1 and Trial-2). Both trials use the same stimulus location and stim intensity. Superposition: comparison of optical signals from ROI-1 (black) and ROI-2 (red) reveals notable differences in the voltage transient half-width. **(B)** Voltage map upon stimulation at Stim-1. Red indicates high, while dark-blue indicates a low amplitude of the optical signal. We selected a scale on which the first Peak reaches the “red” range. Four time points (frames) are selected for display of the spatial distribution of compound (population) voltage. Black circle (hot) marks an area with the greatest depolarization within that time point. White circle (cold) marks the least depolarized area of the brain slice surface. **(C)** Same as in **(B)**, except a new stimulation site (Stim-2) within the same cortical column as in **(B)**.

In this study, synaptic stimulations were delivered in cortical layer 1 (L1), while the EPSPs were recorded in two ways: (a) electrically at the cell body (Figure 2A, patch), and (b) optically at selected regions of interest, ROIs (Figure 2A). The extracellular (synaptic) stimulation protocol employed two triplets of synaptic pulses, at 8.3 and 83 Hz, respectively (Figure 2A, Train-1 and Train-2). In multiple experimental trials, we observed stable voltage responses at both recording locations: (a) electrically at the cell body and (b) optically at ROIs (Figure 2A, compare *Trial-1* to *Trial-2*). Four factors enforced stable and reliable voltage responses in multiple experimental trials: (1) electrical quiescence of a brain slice preparation (lack of sensory inputs, brain states, or spontaneous activity); (2) absence of mechanical disturbances (breathing, heart rate, etc.); (3) patterned stimulation; and (4) temporal and spatial averaging. Each trace in the figure display is a product of temporal averaging (4 sweeps) and spatial averaging (37 pixels inside ROI). In all pyramidal cells tested in this way (*n* = 13), the temporal summation efficacy of the three Peaks at 8.3 Hz (Train-1) (amplitude ratio between the third and the first Peak), was stronger in the population (compound) optical signals (Figure 2A, ROI-2, *strong summation*), but less pronounced in the intracellular recordings (Figure 2A, patch). Namely, the membrane time constant of pyramidal cells, which is about 25 ms^1^ is too short to allow for efficient summation of evoked EPSPs at 8.3 Hz–120 ms inter-stimulus interval (Figure 2A, patch, *weak summation*). What then causes a strong summation of the optical signal? One possible reason is a slow decay dynamic of the GEVI used (chi-VSFP). Slow decaying optical indicators exaggerate temporal summation, or generate an impression of the ongoing summation of electrical signals when there is none (Zhu et al., 2021).

Activation of the neuropil surrounding the pyramidal cell of interest was analyzed using a page display with color-coded pixel intensities. In this mode, one can observe an instantaneous optical signal amplitude simultaneously across the entire visual field (Figure 2B). Images are generated by: (a) selecting one frame from the time sequence (data video), (b) subtracting one frame obtained before the stimulation (for baseline subtraction), and (c) amplitude scaling according to the multi-color scale shown on the side of the optical trace (Figure 2B, red trace). In this color scale, red indicates maximal and dark blue indicates minimal amplitudes. In principle, one can analyze any frame from the sequence of 3,000 frames acquired in each experimental trial. In Figure 2B, we analyzed four instantaneous frames (Frame-B1 to Frame-B4) representing four time points of interest. The time points are marked by black vertical lines transecting the red optical trace (Figure 2B). The voltage map obtained at the first Peak (Figure 2B, first Peak) reveals that voltage activity occurred on the entire surface of the visual field, except in the lower right corner (Frame-B1). In other words, the stimulus at location “Stim-1” inside L1 was unable to excite the lower right corner of this brain slice. The Peak of excitation (red) occurred on the left side of the apical trunk, at the border between L2/3 and L4 (Frame-B1, black circle, “hot”).

The third and the fourth Peak produced remarkably similar voltage maps (Frame-B2 and Frame-B3). In comparison to the first Peak (Frame-B1), here the voltage maps showed a significant growth of the red zone to engulf the entire span of the “left” cortical column, from L1 to L5. Because the third and the fourth Peaks are endowed with higher amplitudes than the first Peak, in the latter Peaks (Frame-B2 and Frame-B3) we also observed enlargement of the green depolarization zone to include the previously “cold” lower right corner. The most exciting finding of this experimental series is that during a compound plateau potential (Figure 2B, red trace, *Plateau*), the voltage depolarizations dwell (sustain) in the same region in which we detected the strongest sensitivity to the first synaptic pulse (first Peak). Compare Frame-B1 and Frame-B4 – the black circle is marking the exact same section of the brain slice. The axons of neurons stimulated at location “Stim-1” project to the area marked by the black circle in Frame-B1 and here release glutamate on repeated (three pulses) high-frequency (83 Hz) stimulation. The released glutamate is not cleared from the release site quickly enough, causing a sustained depolarization of dendritic branches (plateau). As a result of this biological process, the GEVI voltage imaging gives away a long lasting optical signal restricted to the neuropil marked by the black circle in Frame-B4.

Staying in the same cortical column, but changing slightly the position of the extracellular stimulation electrode, down and right, from location Stim-1 to location Stim-2 (Figure 2C), produced a new set of interesting conclusions. First, the most sensitive area shifted slightly to the right (following the electrode) and now included both sides of the neuron’s apical axis (Figure 2C, Frame-C1, red). However, the most sensitive area discovered with Stim-1 location (Figure 2B, Frame-B1, black circle), again turned red despite the new stimulus location, Stim-2 location (Figure 2C, Frame-C1, black circle). Second, large amplitude Peaks (third and fourth) caused the activation of the entire cortical column on the left side of the patched pyramidal cell (Figure 2C, Frame-C2 and Frame-C3), as previously seen with synaptic pulses delivered at location “Stim-1.” Third, with the new stimulation location (Stim-2) the lower right corner of the visual field remained “cold,” especially during the plateau potential (Figure 2C, Frame-C4, white circle). Interestingly, despite a slightly different position of the stimulation electrode (from Stim-1 to Stim-2), the “hot” area during the plateau potential remained at the L23 and L4 border, approximately 250 μm lateral to the apical dendrite (Figure 2C, Frame-C4, black circle). Overall, these data suggest that population GEVI imaging may be used to increase the information-yield of the whole-cell experiments in brain slices, by tracking depolarizations on the surface of a brain slice.

### AVV-Delivered GEVI (ArcLightD)

**FIGURE 3:**
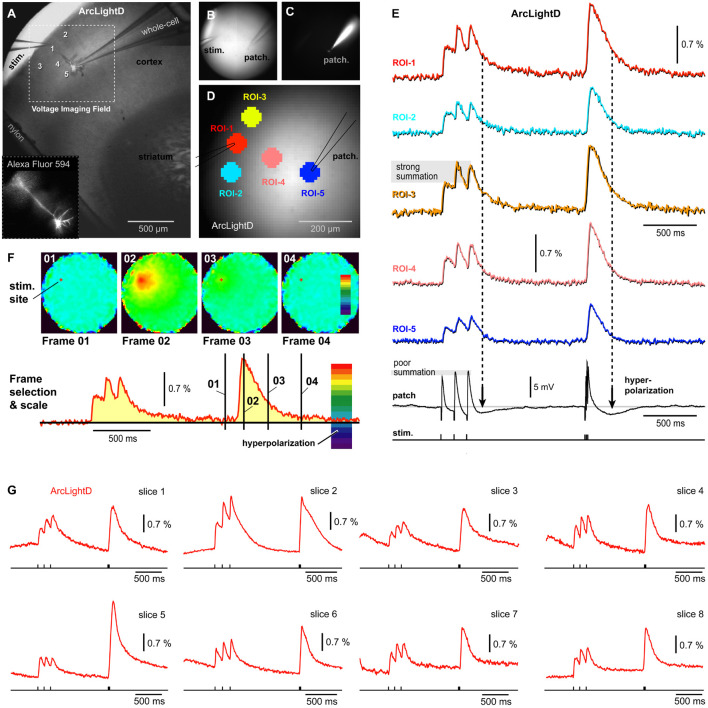
Single cell intracellular recording and population voltage imaging using a GEVI called “ArcLightD.” **(A)** A brain slice with stimulation electrode in L1 and patch electrode in L2/3. **(B)** Transmitted light photograph of two electrodes inserted into a brain slice. **(C)** Same as **(B)**, except fluorescence channel, Alexa Fluor 594. **(D)** Same as **(B,C)**, except the fluorescence channel is ArcLightD. Five regions of interest are marked. **(E)** Optical signals from five ROIs obtained simultaneously with the intracellular recording (whole-cell). Period of neuronal hyperpolarizations are marked by vertical dashed lines. Note that the optical signals show no signs of “hyperpolarization.” **(F)** Page display of optical signal amplitudes at characteristic time points of the experimental course marked by black vertical lines on the red optical trace. “Frame 02” shows the precise location of neural elements activated at the Peak of depolarization. “Frame 03” is obtained at maximal hyperpolarization of the patched cell, yet in Frame 03, no pixels turned dark blue. **(G)** Representative optical traces from the ArcLightD experimental series. Each trace is from a different brain slice.

In the next series of experiments, we tested a different voltage indicator (ArcLightD). Compared to other currently available GEVIs, ArcLightD is endowed with a strong brightness, large optical signals, but slower temporal dynamics (Milosevic et al., 2020). In addition to using a brighter and more sensitive GEVI, we also changed the neuron-labeling technique. In the previous series of experiments, chi-VSFP was carried in the genome of transgenic animals and was present at birth. In this experimental series, ArcLightD was introduced into the brain *via* an AAV injection. At birth (P0.5–P1), animal brains were GEVI-labeled using ICV injections of an AAV_ArcLightD. ArcLightD-injected animals (*n* = 3) were sacrificed 30–50 days postnatal and used for preparation of acute brain slices. One pyramidal neuron was patched in L2/3 and filled with a neuronal tracer dye, Alexa Fluor 594 (Figures 3A–C). Synaptic stimuli were delivered in L1, using the same temporal pattern as described in Figure 2 (*Train-1*, three pulses at 8.3 Hz; followed by *Train-2*, three pulses at 83 Hz). The ArcLightD optical signals (Figure 3) were approximately three times greater than the optical signals obtained with chi-VSFP (Figure 2). More specifically, the amplitude of the fourth Peak in the ArcLightD and chi-VSFP recordings was 1.53 ± 0.25% (*n* = 22) and 0.44 ± 0.02% (*n* = 40), respectively. As previously seen for chi-VSFP, here again the temporal summation during synaptic *Train-1* (120 ms interval) was “poor” in the intracellular recordings (Figure 3E, poor summation) and “strong” in optical recordings (Figure 3E, strong summation). Simultaneous multi-site recordings of population voltage signal (Figure 3D, ROIs 1–5) failed to pinpoint a brain slice area with the strongest amount of temporal summation. Instead, a similar degree of summation was observed at multiple sites including the stimulation site (ROI-1), the flanks of the apical tuft (ROI-2 and ROI-3), the mid apical dendrite (ROI-4), or the cell body (ROI-5). These data suggested that GEVI recordings (ArcLightD and chi-VSFP) exaggerate temporal summation of the neuronal voltage transients.

Following the extracellular (synaptic) stimulation, the patched pyramidal cell experiences an obvious hyperpolarization transient (afterhyperpolarization) in the whole-cell recording (Figure 3E, patch). Based on our whole-cell recordings performed in 13 neurons with L1 stimulation, it is very likely that many pyramidal cells in this area simultaneously experience a very similar voltage waveform: depolarizing potentials followed by afterhyperpolarization. Since the amplitude of the voltage population signal depends on the number of simultaneously activated membranes, one may expect that the population signal too would exhibit a negative transient (hyperpolarization). However, this negative voltage transient was not represented in our optical traces (Figure 3E). To eliminate the possibility that a wrong selection of ROI had caused us to miss hyperpolarization occurring in some area of the brain slice, we examined all pixels simultaneously in a page display mode (Figure 3F). In the page displays, the color scales were set in such a way that a dark blue and deep purple section of the scale coded for negative voltages (Figure 3F, red trace, hyperpolarization). Simultaneous analysis of the entire visual field was performed at several characteristic time points, selected on the course of an experimental sweep (Frames 01–04). Here, Frame 01 establishes a baseline fluorescence prior to a biological event (synaptically evoked depolarization). Frame 02 was set at the Peak of the optical signal with an idea that one brain slice area is depolarized while the other area is hyperpolarized at the same moment – this was never the case. Frame 03 was positioned to intercept the Peak hyperpolarization observed in the whole-cell recordings. Finally, Frame 04 was positioned with some time delay from the Train-2 onset, to examine the possibility that some delayed or slow hyperpolarization permeates the synaptically stimulated brain slice. In experiments in which neurons were not patched (*n* = 22), we examined the time point of an expected maximal L2/3 hyperpolarization (250 ms after the beginning of the Train-2), but found no evidence of hyperpolarization in the optical traces (Figure 3G). The examinations of individual frames or observations of the entire movies (3,000 ms) were unable to detect any signs of hyperpolarization in the ArcLightD voltage imaging experiments (*n* = 22).

### Chimeric Voltage Sensitive Fluorescent Protein Population Imaging

**FIGURE 4:**
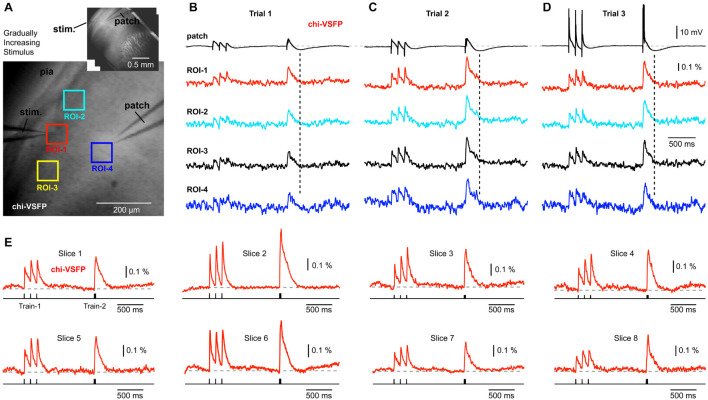
Gradually increasing stimulus intensity failed to reveal hyperpolarizing events in the voltage population signal. **(A)** Brain slice expressing chi-VSFP, with a stimulation electrode in layer 1, and a patch electrode in layer 2/3. Inset: the same brain slice at lower magnification. **(B)** Optical signals from four ROIs obtained simultaneously are aligned with the intracellular whole-cell recording (patch). Vertical dashed line marks the moment of greatest hyperpolarization in the cortical pyramidal neuron. In the following trials **(C,D)**, the intensity of synaptic stimulation was gradually increased until the pyramidal cell fired action potentials (Trial-3). The intensity of synaptic stimulation, which caused pyramidal neuron to fire action potentials, did not result in any detectable hyperpolarization occurring at the population level. **(E)** Examples of synaptically evoked cortical depolarizations obtained in eight brain slices from eight transgenic animals. Chi-VSFP optical signals do not show any signs of afterhyperpolarization occurring upon a synaptic triplet at two different frequencies “8.3 Hz” or “83 Hz.”

Patching L2/3 neurons in brain slices obtained from the chi-VSFP transgenic animals (*n* = 13) has failed to detect any signs of hyperpolarization in the population voltage imaging records (Figures 4A,B). In some experiments, we gradually increased the synaptic stimulation intensity in an attempt to activate inhibitory circuits to deliver stronger inhibitions to the neuronal networks. Stronger synaptic stimuli did change the voltage waveforms in L2/3 pyramidal cells, evident in the whole cell recordings (Figures 4B–D, patch). With stronger extracellular (synaptic) stimulations, we observed intracellular depolarizations of greater amplitudes (compare Trial-1 to Trial-2). Most importantly, an increase in the membrane potential transient of the patched pyramidal cell was accompanied by an increase in the optical signal amplitude at multiple ROIs (Figure 4C). Strong synaptic stimuli eventually caused firing of the patched pyramidal cells (Figure 4D), but the optical signals did not yield any signs of ongoing hyperpolarizations (dashed vertical lines) anywhere on the slice surface.

Establishing a whole-cell recording requires several labor steps including: a search for neurons, micromanipulation of the patch electrodes, making a gigaohm seal, and breaking into the cell. These steps take some valuable time, lifetime, from the brain slices residing in recording chambers. We could work with significantly fresher brain slices if the whole-cell recordings were omitted from the experimental design. In 40 brain slices from 14 animals, we omitted the patch electrode part, and we quickly recorded synaptically evoked optical signals using an identical stimulation paradigm explained in Figure 2 (Train-1 followed by Train-2). In Figure 4E, we display the best traces from eight such experiments, side-by-side. Each trace is an average of 4 sweeps (temporal averaging) and 37 pixels (spatial averaging), to improve signal quality. A significantly shorter time spent in the recording chamber, combined with very strong and cortex-wide expression of chi-VSFP exclusively in pyramidal cells, did not improve our ability to observe negative voltages in the population imaging mode (*n* = 40). Overall, GEVI population imaging with two different indicators, ArcLightD and chi-VSFP, and two different labeling strategies [(a) ArcLightD – AAV injection; and (b) chi-VSFP – transgenic animal], and two different expression patterns [(a) ArcLightD – all neurons; and (b) chi-VSFP pyramidal neurons only], failed to detect any signs of negative population voltages occurring anywhere on the surface of a cortical brain slices receiving synaptic stimulation.

### Archon1 Population Imaging

**FIGURE 5:**
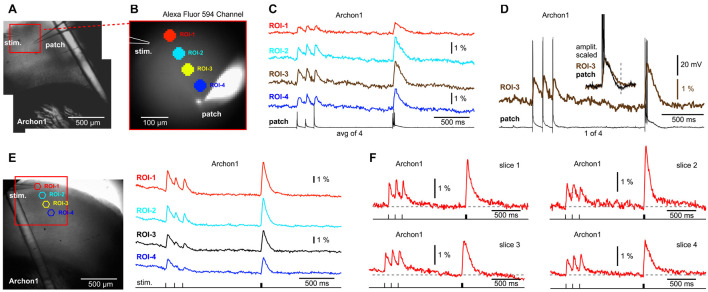
No signs of network hyperpolarizations while imaging with a GEVI called “Archon1.” **(A)** A brain slice expressing Archon1, with stimulation electrode in layer 1, and patch electrode in layer 2/3. **(B)** Photograph of the visual field in the AF594 channel shows fluorescent dye in the: (a) patch pipette, (b) neuron cell body, and (c) apical dendrite of the patched cell. **(C)** Optical signals from four ROIs (obtained simultaneously) are aligned with the intracellular whole-cell recording (patch) sampled at 1 kHz and averaged four times. Voltage scale is provided in **(D)**. **(D)** ROI-3 signal is aligned with a single sweep whole-cell recording. Inset: the optical and electrical signals are cropped, scaled, and superimposed to emphasize a good match of the signal’s slow component. Dashed vertical line marks hyperpolarization in the whole cell recording. **(E)** Same as **(A)**, except different animal. A patch recording was not obtained. **(F)** Examples of synaptically evoked cortical depolarizations obtained in four brain slices from two animals. The “Archon1” optical signals show no signs of afterhyperpolarization upon a synaptic triplet stimulation performed either at “8.3 Hz” or “83 Hz.”

The next GEVI variant examined, Archon1, is more sensitive and exhibits faster ON and OFF kinetics than ArcLightD or chi-VSFP on side-by-side measurements performed by a group that was not involved in developing these three GEVIs (Milosevic et al., 2020). We assumed that a faster voltage indicator, with a shorter decay time, would improve our ability to detect synaptically evoked hyperpolarizations in cortical brain slices. Animals were ICV injected with AAV_Archon1 at birth (P0.5–P1) and sacrificed 30–50 days later for preparation of brain slices. As with previous experimental designs, here again one electrode was used for stimulation (stim.) and the other electrode was filled with Alexa Fluor 594 and used for whole-cell recordings (Figures 5A,B, patch). Extracellular (synaptic) stimulation delivered at L1, produced depolarizations in the cell body of the patched pyramidal cell (Figures 5C,D, patch), but also in the optical ROIs distributed along the apical axis of the patched pyramidal cell (Figures 5C,D, ROIs 1–4). At multiple sites, the Archon1 population voltage imaging failed to detect any signs of a negative optical signal occurring simultaneously in ensembles of neurons (Figure 5C, ROIs 1–4). In six Archon1-labeled brain slices, from two animals, we performed extracellular stimulations without patching the cells. Figure 5E shows a multisite population voltage imaging data with the best signal quality in the Archon1 series. In this experiment, the fourth Peak’s amplitude was ∼6% (Figure 5E, ROI-1), which is ∼4 times better than the average fourth Peak signals typically obtained with ArcLightD, and ∼9 times better than the average fourth Peak signals in chi-VSFP traces. The average amplitude of the fourth Peak in the Archon1 experimental series was 2.31 ± 0.66% (*n* = 6), which was ∼2 times better than the average fourth Peak signals typically obtained with ArcLightD, and ∼5 times better than the average chi-VSFP traces. It is important to emphasize that our optical filters were optimized for ArcLightD and VSFP, but were not optimized for Archon1 (see section “Materials and Methods”), thus it is likely that the Archon1 signal quality could improve further with the use of a 637 nm laser illumination (Piatkevich et al., 2019).

Arguably, the most interesting finding of the Archon1 experimental series was related to the temporal summation of voltage transients. To explain this point, we display the best traces from four slices (Figure 5F). Unlike the ArcLightD series (Figure 3), or chi-VSFP series (Figure 4), in the Archon1 experiments, the Train-1 synaptic stimulation produced modest temporal summations in optical transients (Figure 5), similar to a poor temporal summation observed intracellularly in our study (Figures 3, 4). In summary, Archon1 is faster than ArcLightD or chi-VSFP. With Archon1 imaging, the temporal summation of synaptically evoked network transients was weak. Therefore, the slow OFF kinetics of the GEVI variants *ArcLightD* and *chi-VSFP* appears to exaggerate the temporal summation efficacy in the population voltage transients.

### di-4-ANEPPS Population Imaging

**FIGURE 6:**
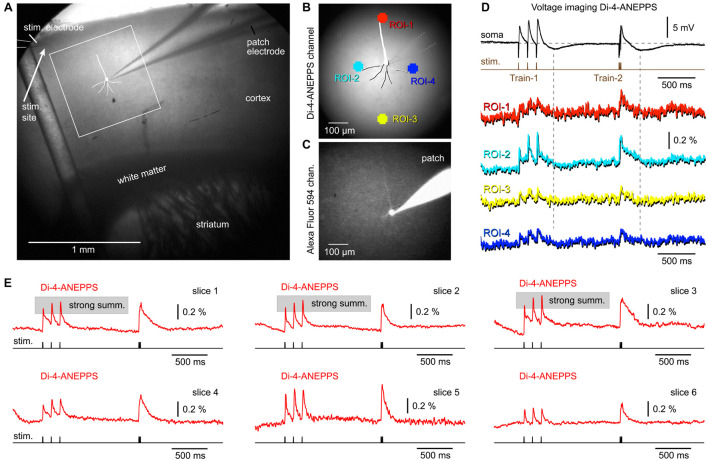
Combined whole-cell recordings and voltage-sensitive dye imaging. **(A)** Brain slice from a wild-type mouse is captured by infrared video microscopy. Drawing marks a cortical pyramidal neuron with the whole-cell patch electrode attached. **(B)** Regions of interest (ROIs) are marked around the patched cell. **(C)** The same cell as in AB, observed in the “594 nm” fluorescence channel. The patch electrode is filled with Alexa Fluor 594. **(D)** An optical signals obtained simultaneously from four ROIs are aligned with the intracellular membrane potential obtained in the cell body (soma). **(E)** Examples of synaptically evoked cortical depolarizations obtained in six brain slices from six animals. di-4-ANEPPS optical signals do not show any tangible signs of afterhyperpolarization occurring upon a synaptic triplet stimulation at two frequencies, “8.3 Hz” or “83 Hz.”

Voltage-sensitive dyes exhibit faster ON and OFF kinetics than any of the currently available GEVIs, including ArcLightD, chi-VSFP, ASAP3, or Archon1 (Milosevic et al., 2020). We assumed that a faster voltage indicator, with a shorter decay time, would finally reveal synaptically evoked hyperpolarizations, which occur in cortical pyramidal neurons upon triplets of synaptic pulses (Figures 3, 4, patch). Brain slices of wild-type mice were extracellularly stained with a voltage sensitive dye (VSD), *di-4-ANEPPS*, an extracellular stimulation electrode was positioned in L1 (Figure 6A), and then one pyramidal cell was patched with a micropipette filled with neuronal tracer, Alexa Fluor 594 (Figures 6B,C). Upon triplets of extracellular (synaptic) pulses, the patched pyramidal neuron experienced synaptic depolarizations (excitatory synaptic potentials, EPSPs) followed by a hyperpolarization transient (Figure 6D, vertical dashed lines), which was not reflected in the optical signals anywhere on the surface of a VSD-stained brain slice (*n* = 7). To improve signal and quality of brain slices, we performed a series of VSD imaging experiments without whole cell recordings (*n* = 6). In Figure 6E, we display the best traces obtained in six brain slices from four animals. In the VSD experimental series, we never found any signs of population (network) signaling that overshoots the baseline and makes a negative dent right after a train of EPSPs. According to the literature, a hyperpolarization following a train of EPSPs would be expected upon a strong and synchronized synaptic inhibition (Avoli, 1986; Agmon and Connors, 1992), or strong and synchronized afterhyperpolarization occurring in the somata of pyramidal neurons (Alger and Nicoll, 1980), occurring in the population of neurons. These data suggest that the slow OFF kinetics of the GEVI indicators (Figures 3–5) were not responsible for the failure of voltage imaging to detect signs of network hyperpolarizations. A voltage indicator with an excellent OFF kinetics, voltage sensitive dye di-4-ANEPPS, also failed to detect network hyperpolarizations (*n* = 7 with patching, and *n* = 6 without patching). The most likely explanation is that large ensemble hyperpolarizations were not evoked in the current experimental paradigm (preparation + stimulus).

Besides helping with the interpretation of “missing” hyperpolarizations in population responses, the experiments with VSD also helped determine the nature of the “exaggerated” temporal summation in optical traces (Figures 2–4). Using a fast indicator di-4-ANEPPS, we observed many examples of strong temporal summation in the optical signal, causing us to revise our previous conclusion that strong summation in optical traces is entirely due to a slow OFF kinetics of the GEVIs used in the current study (Figures 2, 4). In fact, a significant number of mouse cortices stained with a voltage sensitive dye responded with a strong temporal summation in the voltage-imaging signal (Figure 6E, slices 1–3). Here, the OFF kinetics of the voltage indicator, di-4-ANEPPS is on the order of microseconds (Loew et al., 1992) and the expected degree of an artificial optical signal integration should be weak to none. Bottom-line, the temporal summation in the population voltage imaging experiments is real, it varies between preparations (Figures 3–6), and it is slightly exaggerated by slow indicators, ArcLightD and chi-VSFP (Figures 2–4).

### Photobleaching of ArcLightD, chi-VSFP, di-4-ANEPPS, and Archon1

**FIGURE 7:**
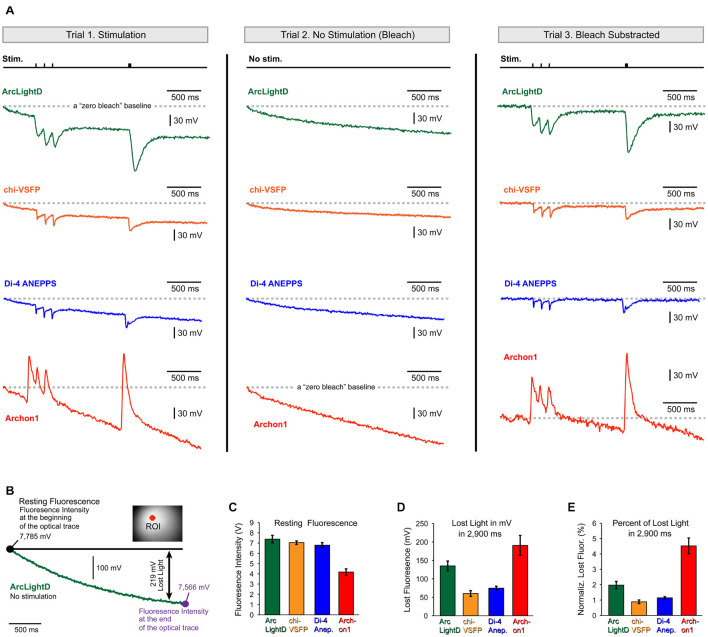
Photobleaching. **(A)** Each voltage indicator, ArcLightD (green), chi-VSFP (orange), di-4-ANEPPS (blue), and Archon1 (red), was analyzed in three steps. The Trial-1 trace is obtained during extracellular stimulation (Stim.). In Trial-2, stimulation was omitted. The Trial-3 trace is a result of subtraction (Trial-1 – Trial-2). A gray dashed horizontal line (“zero bleach” line) indicates a trajectory of an ideal optical trace – no photobleaching. **(B)** A “no stimulation” trial was recorded in a brain slice expressing ArcLightD (green trace). The intensity of the detector photocurrent (converted into millivolts) is shown at the beginning and at the end of a 3 s-long optical trial. Double arrow marks the difference between “ideal no bleaching” indicator (black full line) and the actual recorded trace (green); in this example it is 219 mV. **(C)** Average resting fluorescence measured at the beginning of the optical trace. **(D)** Average lost light measured at the end of the optical trace. **(E)** The lost light measured at the 2.9 s time point is expressed as a percent of the resting fluorescence intensity measured at the beginning of the optical trace. ArcLightD (green, *n* = 22 optical traces), chi-VSFP (orange, *n* = 41), di-4-ANEPPS (blue, *n* = 33), and Archon1 (red, *n* = 8).

Some of the conclusions in the present study may be directly affected by the photobleaching artifact. For example, the neuronal afterhyperpolarization is characterized of a small amplitude (much smaller than the EPSPs we regularly detect, Figures 3–6) and very slow dynamics (much slower than the EPSPs). Both, the small amplitude and slow dynamics can easily be “contaminated” or obscured by unstable baseline in optical signals. The voltage imaging methods suffer a major problem of photobleaching, which creates unstable baseline conditions (Salzberg et al., 1977). Per each new experimental configuration, including the switch to a new voltage indicator, the severity of photobleaching should be carefully evaluated. Here, we evaluated photobleaching in three steps. In the first step, we recorded cortical optical signals with extracellular stimulation turned ON (Figure 7A, Trial-1, Stimulation). In the second step, we recorded optically from the same visual field (same illumination intensity) with extracellular stimulation turned OFF (Figure 7A, Trial-2, No Stimulation – Bleach). Trial-2 produces an optical record of indicator decay with time (photobleaching) that can be used to correct the Trial-1 optical trace. In the third step, we subtract Trial-2 from Trial-1 to arrive at an optical signal void of the photobleaching wobble along the baseline (Figure 7A, Trial-3, *Bleach Subtracted*). The same the-step procedure is displayed for four fluorescent voltage indicators, ArcLightD, chi-VSFP, di-4-ANEPPS, and Archon1 (Figure 7A).

Quantification of the photobleaching effect was performed at two characteristic time points: at the beginning and at the end of a 3 s-long optical trace (Figure 7B, large black dots). The first time point obtained at the beginning of the trace is equivalent to the so-called resting fluorescence intensity (F). It was measured using the raw amplitude of the detector photocurrent (converted to millivolts *via* a current to voltage converter). The average F in the ArcLightD, chi-VSFP, di-4-ANEPPS, and Archon1 experiments was 7.38 ± 0.37 V (*n* = 22); 7.04 ± 0.17 V (*n* = 22); 6.79 ± 0.25 V (*n* = 33); and 4.17 ± 0.31 V (*n* = 8), respectively (Figure 7C, Resting Fluorescence). The second time point was measured exactly 2.9 s after the first. We calculated the amount of lost light in 2.9 s as a difference in F measured at the beginning and F measured at the end of an optical trace (Figure 7B, Lost Light). We found that the average “Lost Light” in the ArcLightD, chi-VSFP, di-4-ANEPPS, and Archon1 experiments was 135 ± 13 mV (*n* = 22); 60 ± 7 mV (*n* = 41); 74 ± 5 mV (*n* = 33); and 190 ± 27 mV (*n* = 8), respectively (Figure 7D, Lost Light). In experiments with large values of “*Resting Fluorescence*,” one typically observes a stronger photobleaching, which would result in greater values for the “*Lost Light*.” To determine if a large difference in F had biased our photobleaching measurements (Lost Light), we calculated the percentage of lost light in respect to the F measured at the beginning of the trace. We found that ArcLightD indicator typically loses 1.97 ± 0.2% of its fluorescent output in 2.9 s of constant wide-field illumination (Figure 7E, ArcLightD). For the same duration of constant wide-field illumination, chi-VSFP, di-4-ANEPPS, and Archon1 had lost 0.88 ± 0.1% (*n* = 41), 1.14 ± 0.1% (*n* = 33), and 4.52 ± 0.5% (*n* = 8), respectively. We determined that the absolute decline of indicator fluorescence in mV (Figure 7E) and the relative decline of fluorescence (in percent of initial F) were very similar among the four indicators tested (compare Figure 7D against Figure 7E). It appears that Archon1 had bleached at a rate of approximately 4% per 3 s of a wide-field illumination.

### Temporal Summation

**FIGURE 8:**
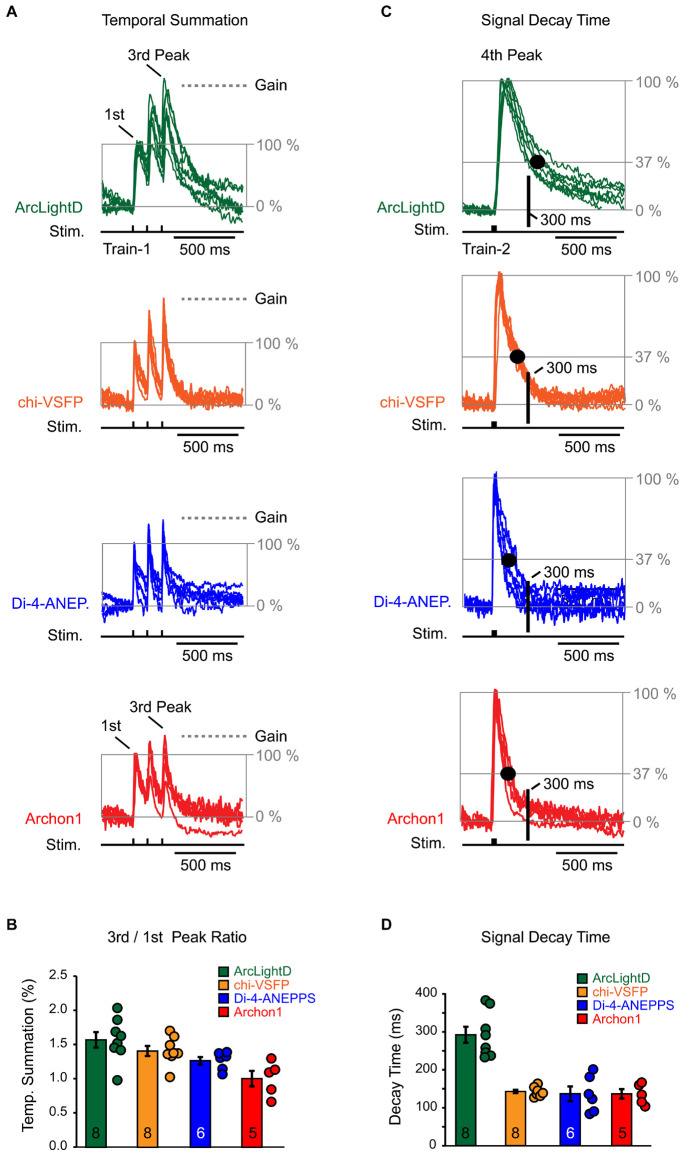
Temporal summation and signal decay time. **(A)** Optical signals were evoked by repetitive synaptic shocks (Stim.) of identical intensity (Train-1 at 8.3 Hz). Synaptically evoked optical traces were amplitude scaled in respect to the first Peak (normalized) and superimposed. The baseline is set at 0% and the first Peak is set at 100%. The gray horizontal dashed line (“*Gain*”) indicates the amount of amplitude change due to temporal summation – an increase in the third Peak in respect to the first Peak. The scaling and superposition of traces was performed for each of the four voltage indicators used in the study, ArcLightD (*n* = 8), chi-VSFP (*n* = 8), di-4-ANEPPS (*n* = 6), and Archon1 (*n* = 5). **(B)** The markers represent an amplitude ratio third Peak/first Peak in individual optical traces. The bars represent mean + SEM. The number of data points used is printed on the bars. **(C)** Synaptically evoked optical traces (Train-2 at 83 Hz) were amplitude scaled in respect to the “fourth Peak.” The amplitude decline down to 37% is marked by the gray horizontal line “*37%*.” Black dot marks the time point at which the optical signal decayed to 37%. Another time point of interest, occurring 300 ms after the stimulus onset, is marked by the thick black vertical line labeled “*300 ms*.” This time point (∼300 ms) corresponds to the hyperpolarization Peak in the whole-cell recordings. **(D)** The markers represent signal decay time (in ms) for each optical trace. The bars represent mean + SEM. The number of data points used is printed on the bars.

We used an identical experimental paradigm throughout the study, which allowed us to compare voltage waveforms obtained with four well-established voltage indicators: ArcLightD, chi-VSFP, di-4-ANEPPS, and Archon1. Representative optical recordings were aligned in respect to the onset of an extracellular (synaptic) stimulation and they were then amplitude-scaled to the first Peak. The first Peak is a network optical signal in response to the first stimulation pulse (Figure 8A, first Peak). With the amplitude of the first Peak set at 100%, we can ask by how much did optical signal increase as we progress from the first to the third event along the synaptic stimulation train. The amplitude difference between the first and the third Peak represents an amplitude gain due to temporal summation of the evoked network responses (Figure 8A, Gain). We found that in voltage imaging experiments using ArcLightD, the temporal summation was 157 ± 11% (*n* = 8). In experiments using chi-VSFP, di-4-ANEPPS, and Archon1, the efficacy of temporal summation was 141 ± 7% (*n* = 8), 127 ± 5% (*n* = 6) and 101 ± 11% (*n* = 5), respectively (Figure 8B). These data indicate that the slowest voltage indicator, ArcLightD, exhibited the greatest efficacy of temporal summation. In the smallest data set, Archon1, two out of five data points showed a decrease in the third Peak amplitude, which brought the average to 101%. We think that this result would not persist if the sample size in the Archon1 data set were enlarged. Nevertheless, a consistent temporal summation obtained with three indicators (ArcLightD, chi-VSFP, and di-4-ANEPPS) indicates that the network responses actually summate in time. Two biological factors likely constitute temporal summation of these population signals: (1) paired pulse facilitation resulting in stronger membrane depolarizations and (2) recruitment of new dendrites on the subsequent events.

### Signal Decay Time

A slow decay time of an optical voltage indicator might interfere with the recording of rapid subsequent events; i.e., the negative potentials (hyperpolarization) that immediately follow the evoked depolarizing potentials detected in the whole-cell recordings (Figures 3–6). To provide a numerical description of the indicator’s OFF dynamics, we used the fourth optical Peak induced by Train-2 (three synaptic pulses at 12 ms interval, 83 Hz). Representative optical recordings of the fourth Peak were aligned in respect to the onset of an extracellular stimulation, and then they were amplitude-scaled (normalized to 100%). We measured the amount of time from the stimulus onset to the point at which the optical signal amplitude decayed to 37% of its Peak value (Figure 8C, large black dot on the decay phase of the voltage transient). We found that a voltage indicator, ArcLightD, exhibited the longest decay, followed by the voltage indicator, chi-VSFP (Figure 8D). More precisely, it takes on average 291 ± 21 ms (*n* = 8) for the ArcLightD signal and 142 ± 4 ms (*n* = 8) for the chi-VSFP signal to fall down to 37% of their Peak values. The 37% decay time for di-4-ANEPPS and Archon1 was 136 ± 19 ms (*n* = 6) and 136 ± 12 ms (*n* = 5), respectively.

From the whole-cell recordings performed in pyramidal cells, we learned that the Peak hyperpolarization occurs ∼300 ms from the onset of the extracellular (synaptic) stimulus (Figures 3–6, dashed vertical lines). We marked this characteristic time point on the optical traces in the figure displays using a thick vertical line labeled “*300 ms*” (Figure 8C). At the intersection of the “300 ms” vertical line and the decay phase of an optical signal, we detected values that are notably above the baseline. In other words, in the greater majority of voltage imaging traces, the optical signal did not return to the baseline in 300 ms. This explains why small-amplitude hyperpolarizations which occur precisely at this time point (300 ms) are difficult to discern in the optical records.

## Discussion

We combined whole-cell recordings from neocortical pyramidal neurons with simultaneous multi-site voltage imaging of population signals in the neuropil surrounding the patched pyramidal cell. The idea here was to test if such population voltage imaging could increase the information content of an *in vitro* (brain slice) experiment and learn more about a relation between the somatic voltage waveform and the synaptically evoked population voltage waveform (Figure 2). We found that population voltage-imaging can reveal “hidden” structures in the voltage propagation pattern, as synaptic potentials (and other associated potentials: dendritic spikes and action potentials) propagate through a network of cortical neurons. Voltage imaging can identify the area of the brain slice in which the strongest depolarizations occur (Figures 2B,C, Frame-1), areas in which postsynaptic depolarizations are weak (Figures 2B,C, white circles), and the areas in which prolonged depolarizations (plateau depolarizations) dwell long after the experimental stimulus (Figures 2B,C, Frame-4). We also found that synaptic stimulations restricted to the same cortical column, produce similar depolarization waves through the network, although these slight changes of the stimulation sites (from Stim-1 to Stim-2) can alter voltage responses in individual neurons; for example, convert subthreshold EPSPs (Figure 2B) to an action potential (Figure 2C). Obviously, the quality and amount of information that can be extracted from dual whole-cell and population imaging experiments will depend on experimental design. One obvious possibility is to replace the synaptic electrode stimulations with optogenetic stimulations, based on cell-type specific expression of actuators (Willadt et al., 2014).

### The Origin of the Population Voltage Imaging Signals

Population imaging does not resolve single cell fluorescence; instead, the neuronal circuit activity from a population of cells is summed into one (compound) signal. Dendrites of pyramidal neurons contribute by far the largest percentage area of membrane within the stained or labeled region of neocortex; hence, the voltage-imaging signal is dominated by dendritic potentials (Grinvald et al., 1982; Kuhn et al., 2008). A very large portion of the population voltage-imaging signal can be blocked by antagonists of AMPA and NMDA receptors (Petersen et al., 2003; Empson et al., 2015). Between the voltage imaging signal and the simultaneously measured subthreshold postsynaptic potential changes, a strong correlation was found in both amplitude and time course (Petersen and Sakmann, 2001; Petersen et al., 2003). VSD images can be converted into reasonable estimates of subthreshold postsynaptic potentials expected in the dendrites of cortical pyramidal neurons, at least under the experimental conditions involving anesthesia and simple sensory stimuli (Petersen et al., 2003). Also, GEVI responses in the brain slice, especially the smaller ones, were exclusively synaptic in nature (Empson et al., 2015). Overall, there is a wide consensus in the field that voltage-imaging signals are dominated by subthreshold glutamatergic postsynaptic potentials (Grinvald and Hildesheim, 2004), just like the local field potential, LFP (Buzsaki et al., 2012). This is in stark contrast to the calcium imaging methods, which predominantly detect neuronal spiking (action potentials).

In order to validate GEVI optical signal as a correlate of neural activity, researchers simultaneously measured the LFP while imaging the ArcLight cortical response to whisker deflections (Borden et al., 2017). They compared the resulting stimulus evoked responses in the LFP and the evoked GEVI fluorescence and found similar characteristics between the two signals, but only if signal averaging procedure were used. The averaged evoked cortical ArcLight signals were 10–25 ms later than the LFP response, and had a ∼3 times slower decay relative to the LFP decay time. Wide-field recorded GEVI fluorescence is believed to represent a spatial measurement of neural membrane potential, which is fundamentally different from extracellularly recorded LFP, where the relationship between the LFP and the membrane potential is not direct (Buzsaki et al., 2012). While there are aspects of the wide-field voltage imaging (population imaging) that reflect features of the LFP, the voltage imaging data contains different and potentially additional information about cortical activation (Borden et al., 2017).

### Indicator Choice

Four voltage indicators and three types of the neuron labeling strategies were explored here, in an attempt to delineate which features of the evoked optical signals are attributable to real biological processes, and which features are mainly artifacts; caused by poor sensitivity, slow dynamics, or the lack of cell specificity. Each voltage indicator used brought something unique to the experimental setup. First, ArcLight is a bright GEVI, producing large amplitude optical signals. It has been used in a variety of preparations, including *in vivo* population imaging of sensory evoked responses (Storace et al., 2019). In our hands, due to perinatal ICV injection of AAV-ArcLightD, some unspecified populations of cortical neurons were labeled with ArcLightD. Second, chi-VSFP is available as a transgenic animal, with strong, dense, and stable membrane expression at birth, engulfing specifically pyramidal neurons, their dendrites, axons, and cell bodies (Empson et al., 2015). The advantages of transgenic animals have been explained elsewhere (Madisen et al., 2015); here we just want to add that not having to worry about the GEVI expression pattern is an enormous benefit and a labor reducer. Third, Archon1 is a GEVI characterized with large optical signals and fast ON–OFF kinetics. Archon1 too, has been proven in the *in vivo* recordings of neural activity (Piatkevich et al., 2019). These recordings were focused on the activity of individual neurons, while the Archon1 population imaging modality (Figure 5) is less explored. With perinatal (P0.5–P1) ICV injection of AAV-Archon1, some unspecified populations of brain neurons were labeled with Archon1. Finally, di-4-ANEPPS is a very well-known and widely utilized voltage indicator (Loew et al., 1992; Grandy et al., 2012).

### Cell Specificity

Three neuron-labeling strategies were tested on an identical experimental paradigm (L1 stimulation, two triplets of synaptic pulses). First, the Tg_chi-VSFP labeling was highly specific and restricted to pyramidal cells only. Second, the AAV_ArcLightD or AAV_Archon1 neuron-labeling was less specific than that with transgenic chi-VSFP labeling. The AAV labeling affected all cell types present in the subventricular zone at P0. Also, the AAV labeling was less uniform, as we observed patches with variable fluorescence. Finally, the extracellular VSD labeling deposited lipophilic indicator molecules on all membranes in the tissue, including pyramidal neurons, interneurons, astrocytes, microglia, and epithelia. The VSD experimental series incorporated voltage activity of synaptically activated interneurons into the population optical signal, unlike the chi-VSFP series, where optical signals came exclusively from the excitatory pyramidal neurons. Despite this relatively important difference between the contributing cellular elements to the population signal (with or without interneurons), the voltage waveforms from VSD traces were nearly identical to those from chi-VSFP traces (compare Figure 4E vs. Figure 6E. This was an unexpected finding. One explanation is that the current synaptic stimulation paradigm is not ideal for a strong activation of inhibitory circuits. Another, probably more important factor is the dominance of pyramidal cells in the cerebral cortex. Not only that pyramidal cells are ∼4 times more numerous than inhibitory GABAergic interneurons, but their dendritic tree carries significantly more membrane. In population voltage imaging, an optical signal is generated in dendrites. Therefore, on a first approximation, a population voltage-imaging signal belonging to pyramidal cells is more than 10-fold stronger than optical signal emanating from inhibitory GABAergic interneurons. This is probably the main reason why optical signals with and without GABAergic interneuron contributions look fairly the same (compare Figure 4E vs. Figure 6E).

### Network Temporal Summation

Neocortical pyramidal neurons have more or less similar membrane time constant, ∼25 ms, and upon a slow (8.3 Hz) stimulation frequency (120 ms inter-stimulus interval), they show a similarly weak efficacy of temporal summation (Figure 2A, patch, *weak summation*). If all neurons that generate the optical signal summate EPSPs weakly at this slow stimulation frequency, then how can an optical signal summate strongly at the same synaptic frequency (Figure 2A, ROI-2, *strong summation*)? This discrepancy between electrical and optical voltage waveforms (Figure 3E) is probably due to two factors. The GEVI’s recovery time is very slow, much slower than the actual EPSP’s decay phase – compare optical and electrical signal belonging to the third Peak (Figure 2A, Trial-2, arrows). This slowness of the GEVI response causes the subsequent events to start before the previous optical signal returned to the baseline, which is a definition of temporal summation. For example, integrative properties of chi-VSFP combined with slow image sampling frequency may cause this “summation effect” (Akemann et al., 2012).

However, this argument was nulled by the VSD voltage imaging experimental series. VSDs have a microsecond response time (Loew et al., 1992), hence they weakly integrate signals. Yet, the VSD population optical signals often exhibited strong temporal summation (Figure 6E). Next, we turn away from the hypothesis that slow OFF kinetics of GEVIs (artifact) is primarily responsible for the observed strong summation of the synaptically evoked optical signals in cerebral cortex, and we explored a potential involvement of biological processes. It is possible that on the second synaptic stimulus a greater number of dendritic branches and axons are activated compared to the number activated on the first stimulus. An increase in number of active elements in turn increases the amplitude of the compound optical signal. Repetitive electrical events, bursts, are known boosters of signal propagation through cortical networks. Cortical synapses are unreliable at signaling the arrival of single presynaptic action potentials to the postsynaptic neuron. However, bursts are reliably signaled because transmitter release is facilitated. Central synapses can be viewed as filters that transmit bursts, but filter out single spikes (Lisman, 1997). With respect to the current experiments based on the triplets of synaptic pulses (Figure 2, Train-1), a greater number of excitable neuropil elements (dendrites and axons) become active on the second and third synaptic pulse compared to the first pulse.

### Hyperpolarization

Upon a barrage of synaptic inputs, a cortical pyramidal neuron experiences a clear afterhyperpolarization (intracellular recording), which for some reason, is not represented in the GEVI population imaging signal (optical signal). We examined the voltage indicator records in our data sets (ArcLightD, chi-VSFP, Archon1, and di-4-ANEPPS), and found no compelling signatures of synaptically evoked inhibitory potentials in the voltage optical signals. No signatures of negative compound potentials (synchronized hyperpolarization) were found in the regions of interest encompassing either the cell body, or basal dendrites, or apical dendrites, of recorded pyramidal cells. Several factors may be responsible for the week evidence of cortical inhibition in our current population signals:

[i] *Shunting*: In cortical brain slices, inhibition may mostly be of a shunting type, with minimal changes in the resting membrane potential occurring upon GABA release (Fatt and Katz, 1953). Note that IPSPs emerge and increase in amplitude only when experimenters artificially depolarize the resting membrane potential (Agmon and Connors, 1992).
[ii] *Sensitivity*: The voltage sensitivity of GEVI imaging is very poor when compared to patch electrode recordings. Weak and slow hyperpolarizations (e.g., −5 mV if any) can easily be lost in the noise, while stronger and faster depolarizations (e.g. +25 mV) have significantly better chances of emerging above the noise level (Figure 3).
[iii] *Experimental paradigm*: The synaptic stimulation paradigm used in the present study (three pulses at 120 ms interval, or three pulses at 12 ms interval, intensity 135 nA) delivered in cortical L1 may not be an ideal setup for evoking strong compound inhibitory signals. Nakajima and Baker (2018) used hippocampal slices to demonstrate inhibitory population synaptic potentials with GEVI imaging. The choice of both, the biological preparation and stimulation paradigm, may be critical to seeing inhibitory population signals.
[iv] *Sustained dendritic depolarizations*: Regardless of the voltage indicator used, GEVI or VSD, a slow decay phase (long excitatory tail) has been a characteristic of voltage sensitive imaging techniques in the cerebral cortex (Figure 8C). This slow decay from the initial synaptically evoked depolarizing response may not entirely represent limitations of the molecule, but potentially additional physiologically relevant information. It is possible that the prolonged fluorescence response represents prolonged excitation caused by a synaptic stimulus, similar to the sustained depolarization lingering in dendritic branches long after the stimulus (Figure 2B, Frame-B4, black circle, and Figure 2C, Frame-C4, black circle), or similar to post-plateau depolarizations accompanying strong NMDA receptor activations in dendrites (Milojkovic et al., 2007).
[v] *Intrinsic*: An alternative possibility is that the long excitatory tail is an intrinsic cortical optical signal, in part (Grinvald et al., 1986). The slowly rising and long-lasting fluorescence transients that occur in cerebral cortex in response to repetitive synaptic stimulations (Empson et al., 2015) may cancel out the network hyperpolarizations.

At the current state of our experimental setting (voltage indicator + equipment), the optical voltage response of many neurons combined (population voltage signal) does not report well the small and slow hyperpolarization transients occurring in individual pyramidal cells, and this can be attributed to unstable baseline in optical recordings. It is very difficult to correct the bleaching artifact even when the “no stimulus” traces are subtracted from the optical records (Figure 7). The hyperpolarizing potentials have a very small amplitude (in the range of only −5 mV) and they are localized to a small fraction of the neuronal surface (perisomatic membrane mostly). On the contrary, depolarizing signals, such as glutamate-mediated dendritic spikes, may achieve ∼50 mV amplitude in dendritic branches (Gao et al., 2021), and these depolarizing signals may occur in any dendritic branch, across the entire dendritic tree of any pyramidal neuron (Oikonomou et al., 2014). Large amplitude depolarizations in dendrites combined with large active membrane area contained in dendritic branches, allow the depolarizing signals to dominate over the hyperpolarizing population responses (small amplitude and restricted to perisomatic membranes) (Isaacson and Scanziani, 2011).

## Summary

• Pairing of electrical recordings (e.g., whole-cell) and voltage imaging (e.g., GEVI imaging) is useful for studying neuronal circuits.
• Observing the same biological phenomenon (e.g., temporal summation of network responses) with two or more voltage indicators of variable sensitivity and a range of ON–OFF kinetics may significantly improve data interpretation.
• All four voltage indicators used in the present study (ArcLightD, chi-VSFP, di-4-ANEPPS, and Archon1) performed remarkably well in the context of synaptically evoked population signals in acute brain slices.
• While the fast voltage transients (e.g., ensemble EPSP) are faithfully represented in voltage imaging records, the slow voltage transients (e.g., slow hyperpolarization) are markedly contaminated by: (1) slow decay time of the indicator optical response; and (2) imperfect photobleach-correction procedures. The curvature of a photobleaching process and attempts to correct for photobleaching by (a) subtracting a “no stimulus” traces, (b) subtracting exponential fits through data points, or (c) application of digital high-pass filters, still leaves some uncertainty about the presence of slow membrane potential changes.
• In the current experimental paradigm (L1 extracellular stimulation), afterhyperpolarization occurring in many individual pyramidal neurons was not reflected in the ensemble of neurons population voltage signal. It is possible that “lack of hyperpolarization” is due to a mismatch of the stimulation protocol with the underlying physiology, and/or reflects the underlying physiology, in which long-lasting depolarizations with slow decays dominate the membranes of the neuropil.
• What is believed to be a localized “synaptic stimulation” delivered *via* extracellular stimulation electrodes inserted into a cortical L1 actually “lights up” almost an entire cortical column to some degree (Figure 2). Fast multi-site voltage imaging techniques, performed using GEVIs or voltage sensitive dyes, may reveal important missing details pertaining to depolarizations occurring in the cortical neuropil during the experiment. Population voltage imaging performed in coronal brain slices can reveal cortical layers experiencing the greatest amount of depolarization, cortical layers that are not activated in a specific experimental paradigm, segments of cortical layers with and without depolarizations, and areas of cortical neuropil that experience sustained depolarizations long after the cessation of stimuli.

## Data Availability Statement

The raw data supporting the conclusions of this article will be made available by the authors, without undue reservation.

## Ethics Statement

The animal study was reviewed and approved by the UConn Health Institutional Animal Care and Use Committee (IACUC).

## Author Contributions

SA designed the research. MZ and JJ developed the animal colonies. JJ, MZ, AJ, and SA performed the experiments and analyzed the data. JJ, AJ, and SA wrote the manuscript. All authors contributed to the article and approved the submitted version.

## Conflict of Interest

The authors declare that the research was conducted in the absence of any commercial or financial relationships that could be construed as a potential conflict of interest.

## Publisher’s Note

All claims expressed in this article are solely those of the authors and do not necessarily represent those of their affiliated organizations, or those of the publisher, the editors and the reviewers. Any product that may be evaluated in this article, or claim that may be made by its manufacturer, is not guaranteed or endorsed by the publisher.

## Footnotes

1
https://neuroelectro.org/
